# Inhalable microplastics and plastic additives in the indoor air of chemical laboratories

**DOI:** 10.1038/s41370-025-00768-0

**Published:** 2025-03-29

**Authors:** Joel D. Rindelaub, Gordon M. Miskelly

**Affiliations:** School of Chemical Sciences, University of Auckland, Private Bag 92019, Auckland, 1142 New Zealand

**Keywords:** Microplastics, Phthalates, Air pollution, Indoor dust/house dust/dust, Inhalation exposure

## Abstract

**Background:**

While recognition of airborne microplastics is increasing, there are still limited data on the microplastics within the aerosol size fractions most relevant to human inhalation (PM_10_ and PM_2.5_). Additionally, there are concerns that many of the additives used in plastic formulations have endocrine-disrupting properties, which could increase the hazards associated with microplastic exposure.

**Objective:**

To better understand the toxicological risks associated with airborne microplastics, more data are urgently needed on the mass concentrations of both microplastics and the related chemical additives in the air we breathe. Inhalation exposure to plastic-related species is currently uncertain in chemical laboratory workplaces.

**Methods:**

Using a Pyrolysis Gas Chromatography Mass Spectrometry (Pyr-GC/MS) based method, the airborne mass concentrations of both polymeric material and small molecule plastic additives were determined in inhalable air from two indoor locations. This method represents a fast, direct technique that can be used to better standardize airborne microplastic measurements.

**Results:**

The PM_2.5_ and PM_10_ concentrations of seven different polymers were determined, with average plastic concentrations of 0.51 μg m^−3^ for the PM_2.5_ samples and 1.14 µg m^−3^ for the PM_10_ samples. Polycarbonate, polyvinylchloride, and polyethylene had the highest airborne concentrations in the inhalable fraction of air. Simultaneously, the airborne concentrations of plastic additives were determined, with phthalate-based plasticizers having an average concentration of 334 ng m^−3^ across all air samples.

**Impact:**

- Both microplastics and their chemical additives were quantified within the inhalable fraction of indoor air (PM_10_), using a straight forward mass spectrometry technique with minimal sample preparation. This information furthers knowledge on the hazards associated with indoor air exposure, and it presents a useful methodology for the mass quantification of plastic-related airborne pollutants.

## Introduction

Microplastics are a growing environmental concern. Recent studies have detected microplastics in atmospheric samples [1, 2], indicating that inhalation may be an important route of human exposure to microplastics. While a significant portion of research has focused on the sources and environmental fate of microplastics in outdoor settings, a rapidly emerging issue is the presence of microplastics within indoor environments, particularly in the air we breathe. Since individuals in developed nations are estimated to spend ~90% of their lives indoors, e.g. via office work, home life, transport [3], the inhalable fraction of indoor plastics may be a considerable source of exposure.

In addition to detection in the human liver [4], blood [5], feces [6], and placenta [7], microplastics have been found in the lungs and the lung tissue of cancer patients [8, 9]. Particulates less than 10 μm in diameter can enter the airways, with smaller sizes able to penetrate deeper into the lungs [10]. Particle sizes less than 2 μm can deposit in the alveoli, and those less than 0.1 μm have potential to enter cells and/or cross the blood−brain barrier and translocate throughout the body [11]. While the health impacts of microplastic exposure are still uncertain, there is potential for induced oxidative stress [12] and cytotoxicity [13], along with subsequent leaching of chemical additives [14] and/or adsorbed pollutants [15].

Indoor microplastics may originate from a wide range of sources, including textiles and fabrics [16], consumer products [17], and building materials [18]. Understanding the sources, distribution, and characteristics of indoor airborne microplastics is essential for assessing the potential health risks associated with inhalation of these particles. Furthermore, as our understanding of indoor microplastics is still evolving, it is imperative to establish a robust scientific foundation to guide building material research, policy development, and public health interventions.

Previous indoor microplastic work has focused on household dust and fallout experiments [19], with fibers being identified as the most regularly detected plastic morphology [16, 20]. Due to the large sizes of the fibers reported in these earlier studies, the greatest hazard from exposure would be through ingestion rather than inhalation. Critically, more data are needed on the sizes ranges small enough to be inhaled into the respiratory system (<10 μm). Additionally, many early environmental microplastic studies relied on reporting the number counts of microplastics from fallout despite the extremely size dependent nature of this metric [2, 21–27], as microplastic numbers increase dramatically at lower size ranges that are often unreported [1].

Moreover, visual inspection of indoor fallout does not provide either the mass of microplastics or their airborne concentrations, both critical pieces of data when assessing toxicological risk. Previous work relying on visual inspection may be prone for misidentification, due to the inability to identify the chemical composition of samples [21, 28]. Techniques such as μ-FTIR and Raman microscopy can provide polymeric identification, however, throughput issues and size limits can potentially lead to significant biases [29].

Exposure to the additives used in plastic formulations may hold greater health risks from exposure than the polymers themselves [30]. While indoor plastic additives and semi-volatile organic compound concentrations have been determined in indoor air [31–34], many studies have not been conducted in conjunction with airborne microplastic measurements, thus limiting our knowledge on the true health hazards of microplastic pollutants. Plastic additives include compounds like di(2-ethylhexyl) phthalate (DEHP) [35] that may interact with endocrine systems, adding further uncertainty to our knowledge of microplastic exposure hazards.

Airborne microplastic inhalation may occur through occupational exposure, as it is common in many societies to work ~40 h per week. In relation to the chemical research and education sectors, academics/students/staff spend increased time in chemical laboratories, yet limited work has assessed the inhalation hazards of airborne microplastics in these areas [36–38]. To better understand occupational health, there remains a need to quantify the airborne mass concentrations of both microplastics and potential endocrine disrupting additives in respirable air.

With more detailed information needed on inhalation exposure to small polymeric materials and their plastic additives in chemical laboratories, this study sampled indoor air in two locations at the University of Auckland campus. Using a Pyrolysis-Gas Chromatography/Mass Spectrometry (Pyr-GC/MS) technique to quantify the mass of both plastic-related additives and individual polymers present in the inhalable fraction of aerosol particles (particles less than 10 μm in aerodynamic diameter), this study represents an important step forward in understanding the risks associated with indoor air exposure in an occupational setting. Simultaneous quantitative information on common plasticizers, such as phthalates, will lead to more accurate toxicological risk assessments of time spent indoors and may provide insight into sources of indoor microplastics, such as building and textile materials.

## Materials and methods

Indoor PM_10_ and PM_2.5_ air samples were analyzed using a Pyrolysis-Gas Chromatography/Mass Spectrometry (Pyr-GC/MS) technique. PM_10_ represents airborne particulate matter less than 10 μm in aerodynamic diameter while PM_2.5_ represents airborne particulate matter less than 2.5 μm in aerodynamic diameter. The technique allowed for the quantification of both polymer types and plastic additives present in the inhalable fraction of indoor air.

### Sample collection

PM_10_ and PM_2.5_ samples were collected at two different locations on the campus of the University of Auckland City Campus (Auckland, New Zealand) over a 10-week period. Microplastics sizes greater than 10 µm are not reported in this investigation, as this study focused on the inhalable fraction of airborne microplastics. Active sampling was conducted above the benchtop approximately 2 m from the floor. Each particulate matter sample was collected over approximately a 7-day period at one of two locations. Five samples were collected at Location 1 and five samples were collected at Location 2. Location 1 was classified as a “wet lab” containing 3 fume hoods and 4 work benches where chemistry experiments regularly took place. Location 2 was classified as an “instrument room” that contained both gas chromatograph and liquid chromatograph systems where routine chemical analyses were performed. Five PM_10_ samples were collected and five PM_2.5_ samples were collected on select dates from 20 May 2022 to 22 August 2022. Dates were selected when there were periods of high activity in the sampling locations, such as times when students were conducting organic synthesis experiments. Polymer synthesis did not occur in the “wet lab” prior to (or during) air sampling. Procedural blank filters were placed in a covered glass Petri dish next the samples during collection. The procedural blanks underwent the same storage, handling, preparation (such as filter cutting for insertion into pyrolysis cups), and analysis procedures as the sample filters. Positive controls were completed by placing three PVC particles (approximate diameter = 200 µm) within a separate filter and conducting Pyr-GC/MS analysis to confirm polymer recovery. The PVC standard particles had the smallest grain sizes of the polymer standards investigated and thus were at highest risk of loss during the preparation, storage, and handling procedures. While directly spiking blank filters with known polymer concentrations is a common method to create positive controls in airborne microplastic studies [39–41], future work should focus on the capture of aerosolized polymer standards to better quantify the recovery of airborne polymers.

Particulate matter samples were collected onto 37 mm quartz filters (PALLFLEX Tissuquartz^TM^, Pall Life Sciences) downstream of either a PM_10_ personal impactor or PM_2.5_ personal impactor (200 Series, TSI Inc.). Air flow was controlled by a variable flow sampling pump (Zefon Z-Lite IAQ), and the air flow rate (10 L min^−1^) was calibrated using a digital flow meter (Model 4146, TSI Inc). The average air volume collected in each sample was 106 m^3^.

### Sample analysis

Quartz filters samples were cut into quarters: one quarter was directly analyzed using Pyr GC/MS at 700 °C for polymer quantification while another 1/4 was analyzed using thermal desorption at 300 °C for quantification of polymer additives. Half of the quartz filter sample was archived for future analysis. Samples were stored in glass petri dishes in a refrigerator at 2–4 °C for up to 10 days prior to analysis. Stability studies indicated that up to 8% of the additives may be lost during storage, which is reflected in the reported uncertainty values. Experimental information related to the stability study is provided in Supplementary Table S2. Collected filter samples were not completely homogenous. Replicate testing of different filter sections indicated a standard deviation of 32% in analyte response across a filter, a value that is reflected in the reported uncertainties. See Supplementary Table S3 for further information on replicate testing.

Pyr-GC/MS analysis was based on previous work by Fan et al. [1]. Analysis was conducted using a microfurnace pyrolyzer (PY-2020iD, Frontier Labs) coupled with a gas chromatograph (GC-2010, Shimadzu) and a single quadrupole mass spectrometer (GCMS-QP2010, Shimadzu). Pyr-GC/MS has previously been employed in microplastics research to identify distinct polymer types [42, 43]. Instrument parameters are provided in Table 1.

**Table 1:** The instrument parameters used in the Pyr-GC/MS analysis.

Micro furnace pyrolyzer	EGA/PY-3030D FrontierLabs
Pyrolysis purge gas	Nitrogen
Pyrolysis temperature	700 °C
Interface temperature	300 °C
Pyrolysis time	12 s
**Gas chromatograph**	**GC-2010**
Injector temperature	300 °C
Injector mode	Split 50:1
Column	Frontier Labs GC UA5, 5% diphenyl – 95% dimethylpolysiloxane (30 m, 0.25 mm i.d., 0.25 μm)
Carrier gas	Helium
Carrier gas flow rate	1.0 mL min^−1^
Temperature program	70 °C (2 min hold), increase to 320 °C at 20 °C min^−1^ (5 min hold)
**Mass spectrometer**	**GCMS-QP2010S Shimadzu**
Transfer line temperature	300 °C
Ion Source temperature	230 °C
Ionization energy	70 eV
Scan range	29–500 *m/z*

**Fig. 1:**
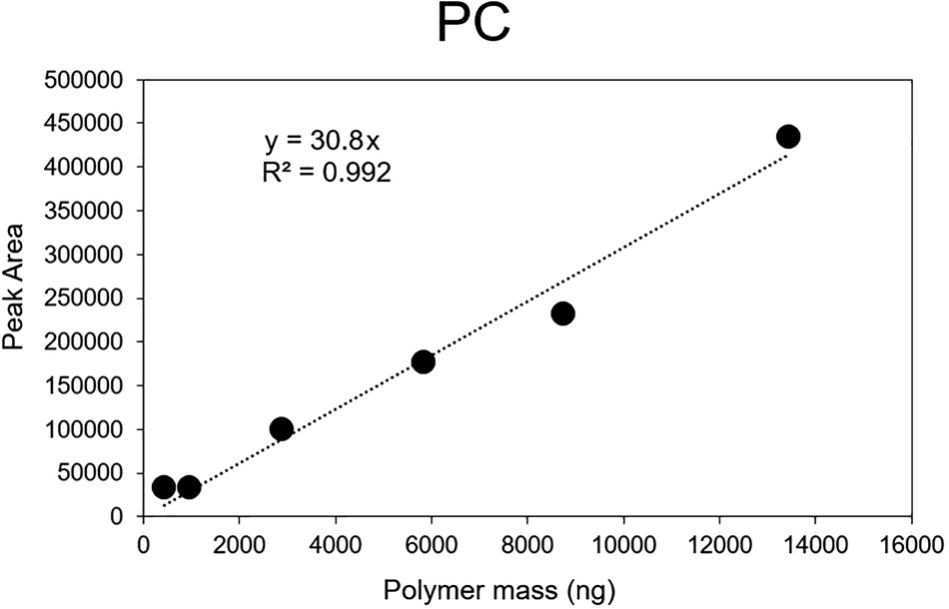
The Pyr-GC/MS calibration curve for polycarbonate (PC). Other calibration curves can be found in Supplementary Fig. S1.

To facilitate polymer quantification at 700 °C, each sample cup was preloaded with 2.0 μL of a 1.776 mg mL^−1^ 5ß-cholanic acid (99%, Sigma-Aldrich) solution in dichloromethane (Macron, AR ACS grade) as an internal standard. The quantifier ion for cholanic acid quantification in the resulting Pyr-GC/MS chromatogram was *m/z* 108, having a retention time of 10.8 min. Calibration curves were performed using authentic external standards [1, 19, 44–46], including polyethylene (PE), polyvinyl chloride (PVC), polypropylene (PP), polyethylene terephthalate (PET), polystyrene (PS), poly(methyl methacrylate) (PMMA), and polycarbonate (PC). The calibration curve for PC is provided in Fig. 1 while the calibration curves for the remaining polymers are provided in Supplementary Fig. S1. All polymer standards were purchased from Scientific Polymer Products, Inc. at 100% purity. Individual polymer standards were created by milling each polymer with calcined sea sand in a ball mil and then transferring a known amount into a pyrolysis cup for analysis, based on previously published methods [1, 44, 46, 47]. The retention times for specific pyrolysis products, both quantifier and indicator, and the relative response factors for each polymer used for analysis are detailed in Table 2. Polymer quantification using Pyr-GC/MS was based on Fan et al. [1].

**Table 2:** The indicator ions, retention times, and relative response factors used in the identification and quantification of polymers in the Pyr-GC/MS analysis.

Polymer	Pyrolysis product	*M (amu)*	Indicator ion (*m/z*)	Relative Response Factor	Method Limit of Detection (ng m^−3^)	Retention time (min)
Polyethylene (PE)	CH_2_ = CH(CH_2_)_7_CH = CH_2_ (C11)	152	83			8.65
	CH_2_ = CH(CH_2_)_10_CH = CH_2_ (C14)^a^	194	83	0.154	7	9.28
	CH_2_ = CH(CH_2_)_11_CH_3_ (C14)	196	83			9.91
	CH_3_(CH_2_)_12_CH_3_ (C14)	198	83			9.23
Polystyrene (PS)	3-butene-1,3-diyldibenzene (styrene dimer)^a^	208	91	2.51	0.7	10.85
	5-hexene-1,3,5-triyltribenzene (styrene trimer)	312	91			14.38
	Styrene	104	104			4.20
Polypropylene (PP)	2,4-dimethylhept-1-ene^a^	126	70	2.67	0.4	3.58
	2,4,6,8-tetramethyl-1-undecene	210	69			8.01
Polyvinyl Chloride (PVC)	Benzene^a^	78	78	14.3	0.5	2.27
	Naphthalene	128	128			7.17
	Indene	116	116			5.83
Nylon6	*ε*-caprolactam^a^	113	113	12.7		7.64
Polymethyl methacrylate (PMMA)	methyl methacrylate^a^	100	100	7.95	0.4	2.51
Polyethylene terephthalate (PET)	Acetophenone^a^	120	105	0.575	4	4.90
	Vinyl benzoate	148	105			6.67
	Ethan-1,2-diyldibenzoate	270	105			13.04
	Divinyl terephthalate	218	175			9.83
Polycarbonate (PC)	Phenol^a^	94	94	3.67	3	5.20
	*p*-cresol	108	107			6.90
	*p*-ethylphenol	122	107			6.08
	*p*-isopropenylphenol	134	134			7.99

The (^a^) indicates the quantifier compound and the relative response factor is the slope of the calibration curve compared to the internal standard (cholanic acid). The relative response factor for Nylon is based on Fischer & Scholz-Böttcher (2019), thus, a method limit of detection could not be provided for this study.

Quantification of plastic additives was accomplished using thermal desorption at 300 °C with tetracosane as an internal standard (1.0 μL of a 117 μg mL^−1^ tetracosane solution in DCM was added to each sample; 99%, Sigma Aldrich). External calibration curves were completed for benzyl butyl phthalate, bis(2-ethylhexyl) adipate, di(2-ethylhexyl) phthalate (DEHP), dibutyl phthalate, diethyl phthalate, dimethyl phthalate, and di-n-octyl phthalate (EPA 506 Phthalate Mix, Sigma Aldrich) as well as triphenyl phosphate (AK Scientific, 99%) and tris(3-chloropropyl) phosphate (AK Scientific, Technical Grade) to assist in compound quantification. With the lack of availability of authentic standards, the reported values of other additives should be considered semi-quantitative with tentative identification. Semi-quantification was performed on triethyl amine, diisopropylethylamine, phthalic anhydride, bis(2-chloro-1-methylethyl) 3-chloropropyl phosphate, tripropylene glycol monomethyl ether (CAS: 20324 - 33 – 8), 2,4,7,9-tetramethyl-5-decyne-4,7-diol (CAS: 126-86-3), diisobutyl phthalate, butyl undecyl phthalate, and 2,2,4-Trimethyl-1,3-pentanediol diisobutyrate (see Results section for more information). Semi-quantitation was based on the response of dimethyl phthalate, which has shown to be an acceptable external standard in plastic-related analyses [48]. Semi-quantitation of bis(2-chloro-1-methylethyl) 3-chloropropyl phosphate was accomplished based on the response of tris(3-chloropropyl) phosphate. Only compounds with a mass spectral similarity score greater than 90% when compared to the NIST spectral database were reported. All reported values were background subtracted from responses detected in the procedural blank filter samples.

## Results

Both polymeric material and small molecule additives were detected in the inhalable fraction of particulate matter (<10 μm in aerodynamic diameter) at concentrations above the procedural blanks in all indoor samples collected. The amount of each analyte detected in the procedural blanks and active samples is provided in Table 3. Airborne concentrations of polymer-related materials were not uniform across the two different indoor locations sampled, similar to previous studies of indoor locations [49, 50].

**Table 3:** The average amount (µg) and standard deviation (µg) of each analyte detected on the filter of both the procedural blanks (*N *= 3) and the active samples (*N *= 10).

Analyte	Avg. amount detected in blanks (µg)	Standard deviation of blanks (µg)	Avg. amount detected in samples (µg)	Standard deviation of samples (µg)
PET	0.98	1.4	3.8	2.1
Nylon	0.034	0.027	0.18	0.20
PC	0.20	0.031	2.0	0.83
PE	1.2	1.4	12.0	3.9
PVC	0.037	0.032	4.6	2.2
PP	0.11	0.094	2.6	4.5
PMMA	BDL	BDL	0.005	0.008
DBP	0.097	0.063	2.0	1.2

*BDL* below detection limit.

### Microplastics

Pyr-GC/MS results at 700 °C yielded positive identification of polyethylene (PE), polycarbonate (PC), polyvinylchloride (PVC), polystyrene (PS), polymethyl methacrylate (PMMA), Nylon, and polyethylene terephthalate (PET). Average airborne mass concentrations for each polymer in the PM_10_ and PM_2.5_ samples are provided in Table 4. The average airborne concentrations of total microplastics was 0.51 μg m^−3^ for the PM_2.5_ samples and 1.14 µg m^−3^ for the PM_10_ samples (an average of 0.83 μg m^−3^ across all samples).

**Table 4:** The average airborne concentrations of the polymers targeted during indoor air sampling.

Polymer	PM_2.5_ conc. (ng m^−3^)	PM_10_ conc. (ng m^−3^)
PE	228 ± 79	256 ± 88
PC	52 ± 18	422 ± 146
PVC	57 ± 20	367 ± 127
PMMA	0.79 ± 0.30	14 ± 5
Nylon	64 ± 22	9.5 ± 3.0
PET	106 ± 37	77 ± 27

**Fig. 2:**
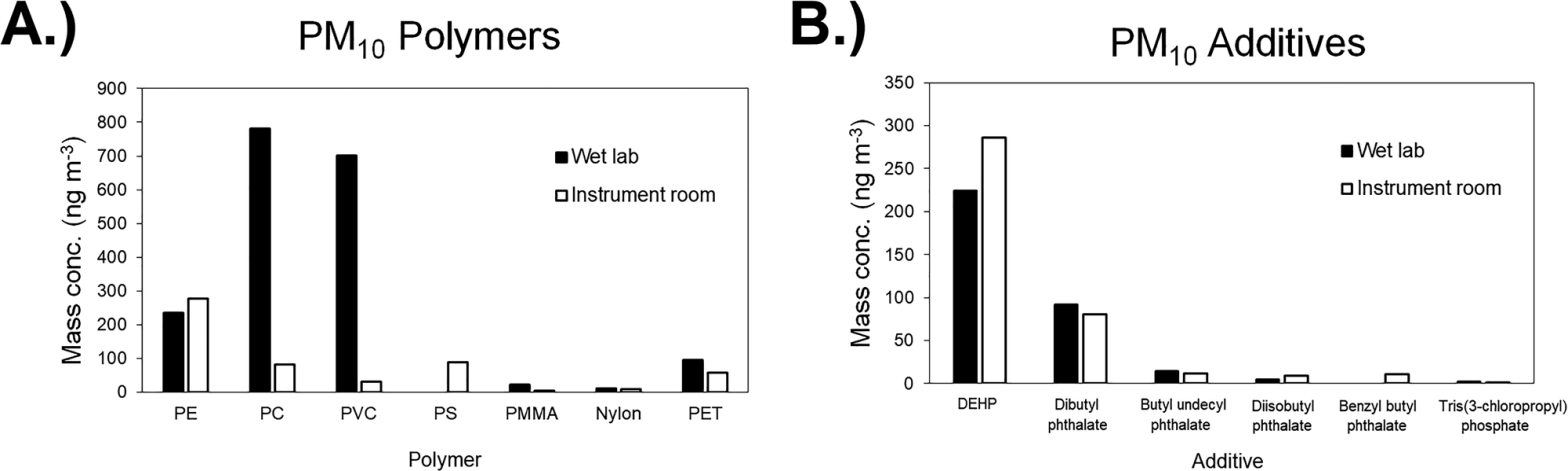
Airborne concentrations of polymers and additives. The average airborne PM_10_ concentrations (ng m^−3^) of (**A**) polymers and (**B**) polymer additives detected in the indoor air samples using the Pyr-GC/MS method.

Airborne polymer concentrations differed between sampling locations, with PC and PVC detected at much greater concentrations in the “wet lab” than in the “instrument room”. Polycarbonate, PVC, and PE were the most abundant polymers detected in this study. The distribution of polymers detected in airborne PM_10_ samples is provided in Fig. 2.

### Plastic additives

Fifteen different additives were either identified or tentatively identified from the thermal desorption analysis at 300 °C using the Pyr-GC/MS method. Seven phthalate-related additives were identified, having an average total concentration of 334 ng m^−3^ across all airborne samples, which is within the range previously reported for indoor air phthalate concentrations (320–4770 ng m^−3^) by Nguyen et al. [31]. The phthalates detected included DEHP, diethyl phthalate, dibutyl phthalate, butyl undecyl phthalate, diisobutyl phthalate, benzyl butyl phthalate, and phthalic anhydride.

Non-phthalate plasticizers were also detected, including common phosphate-based additives triphenyl phosphate and tris(3-chloropropyl) phosphate, which have been previously detected within indoor dust samples [34, 51]. The additives that had the largest concentrations in the airborne particulates are presented in Fig. 2, and complete information on the additives detected is given in Table 5. In addition to compounds commonly used as polymer additives, a surfactant (e.g. from cleaners), an odorant (e.g. cosmetics, liquid soap, cleaners), and a foam suppression agent (e.g. cosmetics) were also detected, indicating that cleaning solutions and cosmetics can also contribute to indoor air quality (Table 5). When plotting airborne polymer additives concentrations against airborne polymer concentrations, two sets of data returned notable correlation coefficients (*r*) and *p*-values (*p*). The airborne concentrations of total phthalates had a strong correlation with airborne Nylon concentrations (*r* = 0.88, *p *= 0.0037) while airborne concentrations of organophosphate esters (triphenyl phosphate, tris(3-chloropropyl) phosphate and bis(2-chloro-1-methylethyl) 3-chloropropyl phosphate) had a strong correlation with airborne PET concentrations (*r* = 0.71, *p *= 0.0493), indicating that each class of additives may have been used in formulations with the respective polymer type (Supplementary Fig. S2).

**Table 5:** The small molecule compounds detected from thermal desorption at 300 °C using the Pyr-GC/MS technique along with their average airborne concentrations.

Compound	Description	PM_2.5_ conc. (ng m^−3^)	PM_10_ conc. (ng m^−3^)
Triethyl amine^a^	Used in making waterproofing agents, and as a catalyst, corrosion inhibitor	7.0 ± 2.5	2.8 ± 1.0
Diisopropylethylamine^a^	Synthetic reagent	0.90 ± 0.30	BDL
CAS: 20324 - 33 – 8^a^	Used in the manufacture of cosmetics, liquid soaps, cleaning formulation, printing and writing inks, and dyes	BDL	0.77 ± 0.27
Phthalic anhydride^a^	Monomer in plastic synthesis	1.9 ± 0.7	2.0 ± 0.7
CAS: 126-86-3^a^	Foam suppression agent in cosmetics	BDL	0.05 ± 0.02
Diethyl phthalate	Plasticizer	53 ± 19	8.4 ± 3.0
Bis(2-chloro-1-methylethyl) 3-chloropropyl phosphate^b^	Flame retardant	4.0 ± 1.4	4.9 ± 1.7
Tris(3-chloropropyl) phosphate	Flame retardant	1.3 ± 0.4	1.3 ± 0.5
Diisobutyl phthalate^a^	Plasticizer	16 ± 6	4.7 ± 1.7
2,2,4-Trimethyl-1,3-pentanediol diisobutyrate	Plasticizer intermediate	1.8 ± 0.6	2.4 ± 0.8
Dibutyl phthalate	Plasticizer	113 ± 40	67 ± 24
Benzyl butyl phthalate	Plasticizer	BDL	4.4 ± 1.7
Triphenyl phosphate	Flame retardant/plasticizer	0.31 ± 0.11	0.74 ± 0.26
DEHP	Plasticizer	127 ± 45	249 ± 88
Butyl undecyl phthalate^a^	Plasticizer	7.2 ± 2.6	15 ± 5

Compounds with an asterisk.

*BDL* below method detection limit of 0.08 ng m^−3^ (based on response of dimethyl phthalate).

^a^Denotes semi-quantitation based on the response of dimethyl phthalate.

^b^Denotes semi-quantitation based on the response of tris(3-chloropropyl) phosphate.

## Discussion

This paper represents a pilot study that provides a straight-forward technique that can used to gather critical information on the airborne mass concentrations of both inhalable polymers and their additives in an occupational environment. Airborne concentrations are much more relevant to determining exposure compared to previous atmospheric fallout measurements. The direct analysis of aerosol filters using Pyr-GC/MS presents a fast and efficient methodology that can be used to minimize sample preparation and the chance for sample contamination, in alignment with previous exploratory studies using direct Pyr-GC/MS techniques for microplastic analysis [52]. Importantly, this method has the potential to quantify polymer additives, such as phthalates, which may hold increased toxicological concern compared to pure polymers [30]. It is important to note that this study could not differentiate particle-bound polymer additives associated with microplastics and those that had adsorbed onto other indoor particulate matter.

Both polymers and additives were found to be location dependent, indicating that indoor airborne microplastics are heterogeneous within a building structure and source dependent, similar to previous findings [50]. While the exact sources of the microplastics detected cannot be determined from this study, the identification of specific polymers can help determine possible origins. The relatively large amount of PC detected in the “wet lab”, for instance, could indicate a higher usage of polycarbonate lab equipment and/or personal protective equipment. The PVC observed could be derived from building materials, and the PE detected could be related to packaging materials. A previous study investigating atmospheric fallout in U.S. households and ambient air also observed relatively large concentrations of PVC and PE [20]. The Nylon and PET polymers observed in this study could result from textiles and clothing, and the relatively small concentrations compared to other polymers detected indicates that primary microplastics expelled from clothing may be too large to inhale (>10 μm).

When considering inhalation exposure of the polymer additives, the average concentration of phthalate species was 334 ng m^−3^. Assuming an adult weighing 60 kg breathes 16 m^3^ of air per day [53], the daily exposure in these locations would result in 110 ng kg^−1^ d^−1^. The European Union’s tolerable daily intake of dibutyl phthalate, an endocrine-disrupting compound that targets male reproduction systems, via ingestion is 10 μg kg^−1^ d^−1^ [54]. It is important to note that inhalation is considered a higher risk dosage form compared to ingestion, and clearance mechanisms from the lungs may be more complicated than digestion processes. In any case, these results represent valuable insight into the potential exposure and toxicological risk associated with indoor air in a working environment that contains chemical laboratories.

Further studies are needed in more indoor locations to better understand exposure to plastic-related chemicals, especially those where humans spend the most time (e.g. households and schools). These studies should expand on the polymers targeted in this study, and they should also investigate low-volatility additives (e.g. Irganox 1010) that may not be easily detected using a GC-based method. Additional studies following the installation of carpets, furnishings, and structural materials are also urgently needed.

## Conclusions

Indoor sampling of PM_2.5_ and PM_10_ revealed the presence of seven different polymers within inhalable air (polyethylene, polyvinyl chloride, polypropylene, polyethylene terephthalate, poly(methyl methacrylate), polycarbonate, and Nylon6) along with the simultaneous detection of common plastic additives. The average concentration of total polymeric material across all samples was 0.83 µg m^−3^ and the average airborne concentration of phthalate species was 334 ng m^−3^. Due to the nature of the sampling methodology, the small molecule additives detected could not be associated with any particular plastic formulation. DEHP was the most abundant plasticizer found in inhalable air, followed by dibutyl phthalate. Phosphate-based additives (e.g. triphenyl phosphate) and those likely to be used in cosmetic and cleaning solution formulations were also detected. The analysis method used provided a direct and efficient method for determining the plastic-related species in inhalable air, helping to minimize the chance for sample contamination and providing a methodology that can be used to better standardize airborne microplastic measurements.

## Supplementary information

Supplemental Information

## Data Availability

Data are available from the corresponding author upon request.
