# Supplementary material for: Inhalable microplastics and plastic additives in the indoor air of chemical laboratories
Source: J Expo Sci Environ Epidemiol. 2025 Mar 29;35(5):785–91. doi: 10.1038/s41370-025-00768-0 (PMC12401729; doi:10.1038/s41370-025-00768-0)

**SUPPLEMENTAL INFORMATION**

**Inhalable microplastics and plastic additives in the indoor air of chemical laboratories**

Joel D. Rindelaub,^1,*^ Gordon M. Miskelly^1^

^1^School of Chemical Sciences, University of Auckland, Auckland, NZ

*corresponding author: j.rindelaub@auckland.ac.nz

**Table of Contents**

**Table S1. Details related to the air samples collected…………………………………... S2**

**Table S2. Storage stability testing……………………………………………………….. S3**

**Table S3. Filter sample replicate testing………………………………………………… S4**

**Figure S1. Calibration curves……………………………………………………………. S5**

**Figure S2. Correlation plots……………………………………………………………… S6**

**Table S1.** Details related to the air samples collected. All sampling dates occurred in 2022.

| **Date started** | **Date finished** | **Location** | **Size fraction** |
| --- | --- | --- | --- |
| 4-May | 12-May | Wet lab | PM_10_ |
| 13-May | 20-May | Instrument room | PM_10_ |
| 19-Jun | 22-Jun | Instrument room | PM_2.5_ |
| 22-Jun | 29-Jun | Wet lab | PM_2.5_ |
| 30-Jun | 7-Jul | Wet lab | PM_2.5_ |
| 25-Jul | 1-Aug | Instrument room | PM_2.5_ |
| 25-Jul | 1-Aug | Instrument room | PM_10_ |
| 1-Aug | 8-Aug | Wet lab | PM_2.5_ |
| 8-Aug | 15-Aug | Instrument room | PM_10_ |
| 15-Aug | 22-Aug | Wet lab | PM_10_ |

**Table S2.** Results from the stability testing of plastic additives stored on quartz filter samples. Quartz filters were spiked in triplicate with 5 µL of a 192 µg mL^-1^ dimethyl phthalate (DMP) solution in DCM (dichloromethane) within a glass petri dishes and then covered with aluminum foil and placed in a refrigerator (Aged samples). DMP was selected as a proxy for all polymer additives as it was the most volatile plasticizer studied and thus had the highest risk for desorption from the quartz filters during storage. After 10 days of storage, the aged samples were injected onto the Pyr-GC/MS along with quartz filter samples freshly prepared with the same amount of DMP (Fresh samples). Prior to injection on the instrument, every quartz filter sample was also spiked with 5 µL of a 53 µg mL^-1^ internal standard (tetracosane) in DCM solution (ISTD). The extracted ion current chromatograms (EICs) of each respective analyte were integrated, with peak areas provided below. To normalize the data, a Response Ratio was calculated by dividing the peak area of DMP by the peak area of ISTD (Response Ratio = DMP/ISTD). The recovery was determined by dividing the average Response Ratio of the Aged samples (N=3) by the average Response Ratio of the Fresh samples (N=3). A blank quartz filter sample was also injected, and all responses from the blank filter were below the method detection limit (BDL).

| **Sample** | **DMP**  **Peak Area (EIC *m/z* 163)** | **ISTD**  **Peak Area**  **(EIC *m/z* 57)** | **Response Ratio** |
| --- | --- | --- | --- |
| Fresh 1 | 1717250 | 674799 | 2.54 |
| Fresh 2 | 947272 | 393365 | 2.41 |
| Fresh 3 | 170531 | 109103 | 1.56 |
| Average |  |  | 2.17 |
| Blank | BDL | BDL |  |
| Aged 1 | 91720 | 70633 | 1.30 |
| Aged 2 | 350139 | 146087 | 2.40 |
| Aged 3 | 1946431 | 838244 | 2.32 |
| Average |  |  | 2.01 |
|  |  |  |  |
| **Recovery** |  |  | **92%** |

**Table S3.** Pyr-GC/MS results from replicate injections of three different quartz filter segments (N=3) taken from a single filter. This was done to examine the distribution of analytes across a collected sample filter. Analyte peak area is provided along with the associated retention time and indicator ion used to create the respective extracted ion current chromatogram for integration. BDL = below method detection limit. RSD = relative standard deviation.

| **Analyte** | **Indicator ion**  **(m/z)** | **Retention time**  **(min)** | **Sample 1**  **(Peak Area)** | **Sample 2**  **(Peak Area)** | **Sample 3**  **(Peak Area)** | **RSD (%)** |
| --- | --- | --- | --- | --- | --- | --- |
| DEHP | 149 | 15.89 | 98270 | 84492 | 95406 | 7.8 |
| PE | 83 | 9.28 | 6830 | 6393 | 5835 | 7.9 |
| Nylon6 | 113 | 7.64 | 10528 | 3673 | 13399 | 54 |
| PS | 104 | 4.20 | 28019 | 25033 | 20667 | 15 |
| PC | 94 | 5.20 | 201686 | 58092 | 64335 | 75 |
| PVC | 77 | 2.27 | BDL | BDL | BDL | N/A |
| PP | 126 | 3.58 | BDL | BDL | BDL | N/A |
| PMMA | 100 | 2.51 | BDL | BDL | BDL | N/A |
| PET | 105 | 4.90 | BDL | BDL | BDL | N/A |
|  |  |  |  |  |  |  |
| **Average** |  |  |  |  |  | **32** |

**Figure S1.** Pyr-GC/MS calibration curves for polyvinyl chloride (PVC), polyethylene terephthalate (PET), poly(methyl methacrylate) (PMMA), polyethylene (PE), polypropylene (PP), polycarbonate (PC), and polystyrene (PS). Due to issues with ball milling and solvent solubility, a calibration curve for Nylon6 could not be completed. Nylon6 was quantified using the reported relative response factor from Klein & Scholz-Böttcher (2017). All Nylon6 data should be considered semi-quantitative.


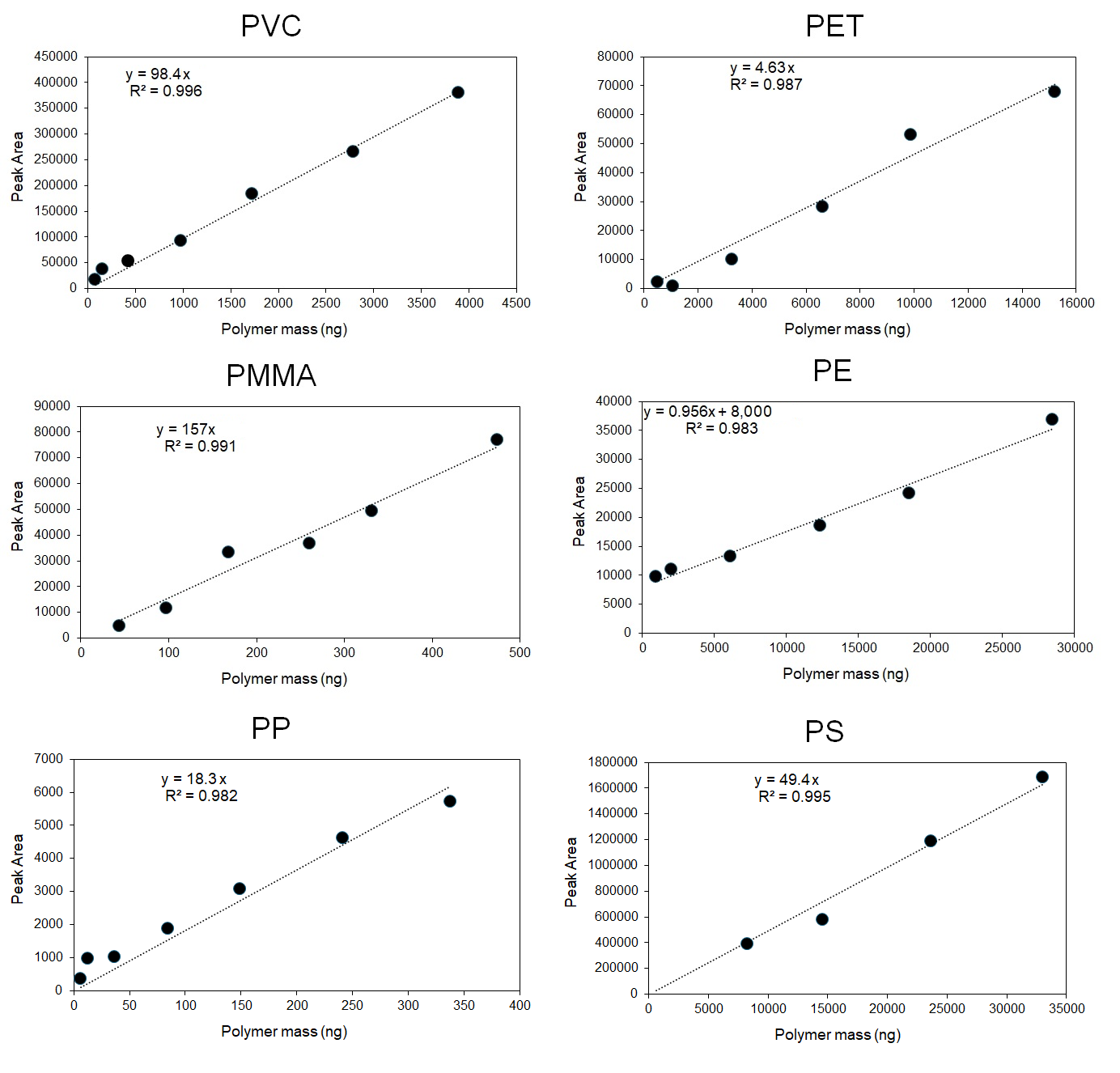


**Figure S2.** The correlation plots between (A.) airborne Nylon6 concentrations (µg m^-3^) and total airborne phthalate concentrations (ng m^-3^) and (B.) airborne polyethylene terephthalate (PET) concentrations (µg m^-3^) and total organophosphate ester concentrations (ng m^-3^). The correlation coefficient (*r*) and p-value (*p*) are provided.


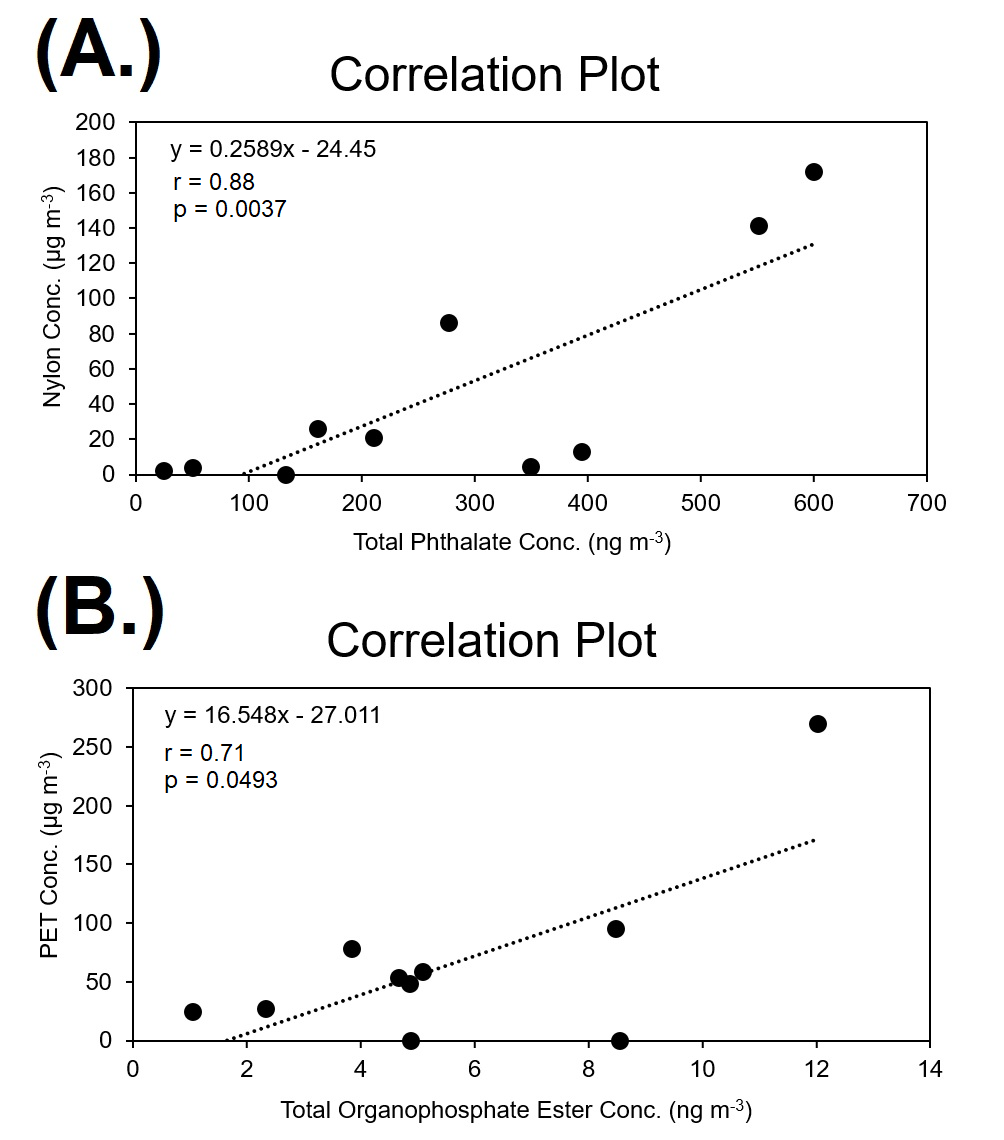

Supplement: Supplementary file 1 — Supplemental Information [file 41370_2025_768_MOESM1_ESM.docx]
